# Insight into schizophrenia: a comparative study between patients and family members

**DOI:** 10.1590/S1516-31802004000600004

**Published:** 2004-11-04

**Authors:** Alexandre Duarte Gigante, Saulo Castel

**Keywords:** Schizophrenia, Awareness, Self concept, Family relations, Social environment, Esquizofrenia, Conscientização, Auto-imagem, Relações familiares, Meio social

## Abstract

**CONTEXT:**

Despite the recognition of the role that sociocultural factors play in the process of acquiring insight, recent research on this issue is scarce.

**OBJECTIVES:**

1) to translate and adapt the Schedule for Assessment of Insight (SAI) to Portuguese; 2) to use a modified version of it to evaluate family members’ insight into schizophrenia; 3) to compare patients’ insight with family members’ insight.

**TYPE OF STUDY:**

Cross-sectional study.

**SETTING:**

Schizophrenia Project Outpatient Clinic (Projesq), Institute of Psychiatry, Faculdade de Medicina da Universidade de São Paulo.

**METHODS:**

40 patients with schizophrenia (Diagnostic and Statistical Manual for Mental Disorders – Fourth Edition — DSM-IV) undergoing outpatient treatment and members of their respective families were interviewed using the SAI and a modified version of this instrument, respectively.

**RESULTS:**

Family members performed better than patients in the total and partial SAI scores [total: 13.0 to 8.75 (p < 0.001); adherence: 3.9 to 3.4 (p < 0.005); recognition of illness: 5.5 to 3.5 (p < 0.001); relabeling of psychotic phenomena: 3.6 to 1.9 (p < 0.001)]. However, when the scores were correlated for each patient-family member pair, the only partial score that had a negative correlation was the relabeling of psychotic phenomena (r = −0.14), while the others had positive correlations (total r = 0.401; adherence r = 0.410; recognition of illness r = 0.422).

**DISCUSSION:**

There was a lack of correlation between the scores of family members and patients regarding the ability to relabel psychotic phenomena as abnormal. This might be understood as a smaller influence of sociocultural factors in this dimension than in other dimensions. The fact that family members were not assessed for the presence of psychopathology is a limitation of this study.

**CONCLUSIONS:**

Different dimensions of insight are not equally influenced by disease and sociocultural factors. The recognition of illness is more strongly influenced by sociocultural factors than the ability to relabel psychotic phenomena as abnormal.

## RESUMO

**CONTEXTO:**

A despeito do reconhecimento da importância de fatores socioculturais na crítica da doença, a literatura recente sobre esse tema é escassa.

**OBJETIVOS:**

1) traduzir e adaptar o Schedule for Assessment of Insight (SAI) para a língua portuguesa; 2) utilizar uma versão modificada deste instrumento para avaliar a crítica dos familiares em relação à esquizofrenia; 3) comparar a crítica dos pacientes com a de seus familiares.

**TIPO DE ESTUDO:**

Estudo transversal

**LOCAL DO ESTUDO:**

Ambulatório do Projeto Esquizofrenia do Instituto de Psiquiatria do Hospital das Clínicas da Faculdade de Medicina da Universidade de São Paulo (Projesq).

**MÉTODOS:**

40 pacientes com diagnóstico de esquizofrenia (Manual Diagnóstico e Estatístico de Transtornos Mentais – Quarta edição – DSM-IV) em tratamento ambulatorial e seus respectivos familiares foram entrevistados pela SAI e por uma versão modificada deste instrumento respectivamente.

**RESULTADOS:**

Os familiares apresentaram melhores notas que os pacientes nas avaliações parciais e total da SAI [total 13,0 e 8,75 (p < 0,001); aderência 3,9 e 3,4 (p < 0,005); reco-nhecimento da doença 5,5 e 3,5 (p < 0,001); reconhecimento dos fenômenos psicóticos 3,6 e 1,9 (p < 0,001)]. No entanto, quando as notas foram correlacionadas entre cada par paciente-familiar, a única nota parcial que teve uma correlação negativa foi o reconhecimento correto dos fenômenos psicóticos (r = −0,14), enquanto os outros tiveram correlações positivas (total r = 0,401; aderência r = 0,410; reconhecimento da doença r = 0,422).

**DISCUSSÃO:**

Não houve correlação entre as notas dos familiares e dos pacientes na habilidade de reconhecer os sintomas psicóticos como anormais. Isso pode ser entendido como uma menor influência dos fatores socioculturais nesta dimensão. A presença de características psicopatológicas e neuropsicológicas nos familiares pode ter influenciado estes resultados. O fato de os familiares não terem sido avaliados para estes déficits é uma limitação deste estudo.

**CONCLUSÃO:**

Diferentes dimensões da crítica da doença não são igualmente influenciadas pela doença e fatores socioculturais. O reconhecimento da doença parece sofrer maior influência dos fatores socioculturais que o componente reconhecimento correto dos fenômenos psicóticos.

## INTRODUCTION

A lack of insight was the most prevalent symptom of schizophrenia found in two seminal international studies, the International Pilot Study of Schizophrenia (IPSS)^1^ and the Classification of Chronic Hospitalized Schizophrenics (CCHS).^2^ In addition, lack of insight has been included among the 12 symptoms that have the highest power to discriminate schizophrenia from other psychoses and depression.^3^ It has been shown that patients with better insight are more likely to present better adherence to treatment.^4,5^ Lack of insight has been correlated with worse outcome,^6^ more admissions,^6^ worse psychosocial functioning,^7,8^ reduced success rates in outpatient treatment of relapses,^9^ and longer interval between the onset of symptoms and the seeking of treatment.^10^

The relationship between insight and psychopathology is controversial. Some authors have proposed that insight is independent of psychopathology^11,12^ while others have found a negative correlation between insight and the general measures of psychopathology.^13^ In a recent meta-analysis of 40 studies, Mintz et al. concluded that there is indeed an association between insight and psychopathology that is weak and mediated by the phase of the illness, as well as by the patient's age at onset of the symptoms. They described a negative correlation between the positive symptoms of schizophrenia and insight that is stronger among patients in the acute phase, and a negative correlation between negative symptoms and insight that is stronger among patients with late onset of symptoms.^14^

Many studies have shown a positive relationship between insight and the depressive symptoms of schizophrenia.^14,15^ Insight has been found to be associated with a higher risk of suicide,^16,17^ but this finding has not been confirmed.^18^ A faint correlation or no correlation at all has been described between insight and neuropsychological deficits.^11,12,19^ A reduction in the overall size of the brain^20^ and atrophy of the frontal lobe^21^ have been shown among patients with insight impairment. Startup^22^ suggested that a relationship between cognitive deficits and insight might only exist among some subpopulations of patients and that there might be stronger influence of psychological and sociocultural factors among those whose cognitive functions but not insight are preserved. Lysaker et al.^23^ found significant evidence to support the idea that there might be two distinct groups with insight impairment: one with deficits of the executive functions, and the other with a strong tendency to use psychological mechanisms of denial of the illness. The role of psychological defenses in the formation of insight has already been recognized by some authors.^24,25^

### The sociocultural context

The scarcity of studies on the social and cultural influences on insight arises in spite of the large number of works on the role played by those factors in the onset, diagnosis, treatment and prognosis of schizophrenia.^26-28^

According to Kirmayer and Corin,^29^ the individual's capacity for self-knowledge stems mainly from social processes, involving the observation of others and the acquisition of ways to describe oneself that are specific to the culture that the individual comes from. Therefore, insight is not a mere act of the patient's self-perception that he or she is ill, but rather a construction that depends on the sociocultural context.

According to Johnson and Orrell,^30^ psychotic patients disagree with their doctors as to their symptoms and illness not only because they are ill, but also because they have a different concept of their experience, which is molded by their sociocultural context. There are standardized ways of thought and action for reporting the experiencing of illness that are guided by the local culture. Patients use these standards, which may differ from the physicians’ standards and from those of patients from different cultures.^31,32^ Cultural influences on the self-evaluation of mental illness are found when groups of psychotic patients from different cultures are studied and compared.^33,34^

In addition to the different conceptions of mental illness, there are other important sociocultural factors. White et al.^35^ found a strong association between the size of the primary group (family and close friends) and insight. They stated, as also postulated by Breier and Strauss,^36^ that broader social contact exerts a normalizing function on the individual that leads to better insight.

Another sociocultural factor that could interfere in the evaluation of mental illness by patients could be stigma, which would be stronger in some specific cultures.^30^ There is evidence that patients’ denial of their illness could buffer the impact of the stigma on patients’ self-appraisal.^37^

Johnson and Orrel^30^ stated that different dimensions of insight are influenced in different ways by psychosocial factors. The ability to relabel psychotic phenomena as abnormal is influenced more by psychopathological factors than by sociocultural ones. Recognition of illness is the variable most affected by the latter factors.

### Evaluation of the attitude of family members regarding the illness

Angermeyer and Matschinger^38^ studied family members’ beliefs regarding the causes of schizophrenia, and showed that they attributed it to biological factors. This perception differed from that of the general population, for whom psychosocial factors were considered to be the most important ones. These authors considered this finding to be the result of the closer contact of family members with the treatment system, as well as their need to deal with the guilt related to the illness. However, in a study by Holzinger et al.,^39^ family members also considered the psychosocial causes to be the main ones. In another study, Angermeyer and Matschinger^40^ came to the conclusion that personal experience with mentally ill patients led to a more positive attitude and to fewer reactions of fear.

The influence of family members on patients’ insight into their illness has been investigated by some authors. Mantonakis et al.^41^ found a positive relationship between negative attitudes towards persons with schizophrenia and low educational level of family members, but no relationship between negative attitudes and higher rates of relapse. Smith et al.^42^ adapted the Scale to Assess Unawareness of Mental Disorder (SUMD) for use among family members, and observed that family members and patients presented similar scores. Family members’ results were better in relation to the attribution of symptoms to the illness. Both groups identified a higher number of symptoms than they were able to attribute to the illness.

### Measurement of insight

There are some standardized instruments designed for assessing insight. All of them approach insight from a dimensional point of view. David^43^ developed the Schedule for Assessment of Insight (SAI), made up of three distinct components: (a) adherence to treatment, (b) recognition of having a mental illness and (c) ability to relabel psychotic phenomena as abnormal. This instrument was validated in a population of 63 schizophrenic patients (45 under outpatient treatment and 18 hospitalized).^44^

Amador et al.^6^ developed the Scale to Assess Unawareness of Mental Disorder (SUMD) comprising the general items of general awareness of having a mental disorder, awareness of the benefits of treatment and awareness of the social consequences of the disorder, in addition to two subscales for evaluating awareness and attribution for each symptom. Training is required for its administration. Fiss^45^ translated it into Portuguese, adapted it to our environment and studied its reliability.

Given the validation data, the brevity of the scale and the possibility of adapting it to family members, we chose to use the SAI in this work.

The objectives of this study were to:

1. Translate and adapt the Schedule for Assessment of Insight (SAI) to the Portuguese language;
2. Use a modified version of this instrument to evaluate family members’ insight into schizophrenia;
3. Compare patients’ insight with family members’ insight.

## METHODS

*Sample*: 40 patients and 40 respective family members were selected from admissions to the Schizophrenia Project Outpatient Clinic (Projesq) of the Institute of Psychiatry, Faculdade de Medicina da Universidade de São Paulo, between January 2000 and December 2000.

The inclusion criteria were:

1. Diagnosis of schizophrenia according to the criteria of the Diagnostic and Statistical Manual of Mental Disorders, Fourth Edition (DSM-IV).^46^
2. Age between 18 and 55 years.
3. Availability of family members to accompany the patients to the interview and for application of the scale.
4. To live the São Paulo metropolitan area.
5. Signing of the Informed Consent Form.

Patients who could not be interviewed because of mutism, negativism or psychomotor agitation were excluded.

One hundred and ninety-five patients were admitted during the aforementioned period; 33 were excluded because they lived outside the defined area or did not have a telephone number for contact. The remaining 162 were randomly sorted and then contacted until we achieved the target sample of 40 patient-family member pairs. One hundred and fifty-four patients were contacted to reach the final sample of 40. Out of the initial 154, 39 (25.3%) were not found (no successful contact after 3 attempts). Seventy-five patients (48.8%) were contacted, but were not included because of another diagnosis (30 patients), dropping out from treatment (18), non-availability of any family member (11), refusal to participate (7), referral to another treatment facility (6), or discharge (3). The 40 patients selected represented 25.9% of the total sample.

### Interview and Instrument

The interviews were carried out by one psychiatrist (the first author). Demographic and clinical data were gathered and the diagnostic inclusion criteria were assessed according to the DSM-IV criteria. The evaluation of insight was carried out using the SAI, for each participant (patient and family member) separately. The scale was a translation of the scale developed by David^43^ and validated in 1997.^44^ The authors of the present study adapted the scale to be applied to family members of the patients (Appendix 1). The interviews were carried out over five months, between February 6, 2001, and July 10, 2001, at Projesq. Patients and family members were interviewed on the same days, except in two cases in which there were intervals of 36 and 78 days.

The SAI comprises three subscales that measure distinct components of insight, namely adherence to treatment, recognition of illness and ability to relabel psychotic phenomena as abnormal. The sum of the scores of the subscales yields a total score of up to 14 points. At the end of the scale there is a supplementary question (hypothetical contradiction) that can add up to 4 points to the total score, which then would reach 18 points. The calculations showed in the results section were made without the supplementary question.

Six demographic variables were recorded for patients and family members: gender, age, ethnicity, marital status, religion and number of years of education. Eight clinical variables were recorded for patients only. These were presence, number and duration of previous hospitalizations, duration of illness, associated psychological treatment, family history of schizophrenia, suicide attempts and age at onset of illness.

Student's t test was used to compare means between the two groups. The chi-squared test was used to compare category variables, and correlations were performed using the Spearman correlation test.

This project was reviewed and approved by the ethics review committee of the Institute of Psychiatry, Faculty of Medicine, Universidade de São Paulo.

## RESULTS

The demographic and clinical characteristics of the two groups are presented in Table 1. One patient had been admitted to the psychiatric hospital and the other 39 were under outpatient treatment at the time of the interview. Regarding antipsychotic medication, 30 patients had no change during the month preceding the interview, 9 had the dosage increased, and one had a gradual change from risperidone to clozapine.

**Table 1:** Demographic and clinical characteristics of the sample of patients and family members

Demographic characteristics	patients (n = 40)	family members (n = 40)	*χ^2^/tt test	p
**Gender % (n)**				
Male	52.5 (21)	20.0 (8)	9.14*	0.002
Female	47.5 (19)	80.0 (32)		
**Age in years (95% CI)**	30.6	50.1	-8.0†	0.001
	(27.5-33.8)	(46.3-53.9)		
**Marital status % (n)**				
Single	75.0 (30)	7.5 (3)		
Married	17.5 (7)	60.0 (24)	39.4*	0.001
Separated	7.5 (3)	15.0 (6)		
Widowed	-	17.5 (7)		
**Ethnicity % (n)**				
White	77.5 (31)	77.5 (31)	0.23*	0.89
Mixed	12.5 (5)	15.0 (6)		
Asian	10.0 (4)	7.5 (3)		
**Religion % (n)**				
Catholic	55.0 (22)	60.0 (24)	4.49*	0.48
Evangelical	15.0 (6)	15.0 (6)		
Buddhist	5.0 (2)	7.5 (3)		
Spiritualist	-	5.0 (2)		
Others	10.0 (4)	2.5 (1)		
No religion	15.0 (6)	10.0 (4)		
**Years of education (95% CI)**	9.2 (8.3-10.1)	9.6 (8.0-11.1)	4.24†	0.673
**Clinical characteristics**				
**Previous hospitalization % (n)**	65 (26)			
**Number of previous hospitalizations,‡ mean (95% CI)**	3.35 (1.7-4.9)			
**Time spent hospitalized over lifetime in weeks,‡ mean (95% CI)**	13.6 (3.9-23.3)			
**Duration of illness in years, mean (95% CI)**	7.9 (5.7-10.1)			
**Present psychotherapy treatment % (n)**	12.5 (5)			
**Family history of schizophrenia % (n)**	52.6 (20)			
**Patients who attempted suicide % (n)**	30 (12)			
**Age at onset of illness in years, mean (95% CI)**	23.1(20.5-25.6)			

‡ *Refers to patients who had already been hospitalized; CI = confidence interval*.

Insight among the patients was not related to the demographic variables of age, gender and number of years of education, or to the clinical variables of age at onset of illness, duration of illness, number and duration of hospitalizations, suicide attempts (n = 12), previous hospitalization (n = 26), or family history of schizophrenia (n = 20). The patients who had the medication changed during the preceding month (n = 11) did not present significant differences in insight into their illness, compared with those who did not have it changed. The subgroup of patients who were under psychological treatment (n = 5) had better insight into the illness (t = −3.64; p = 0.004).

**Figure 1:**
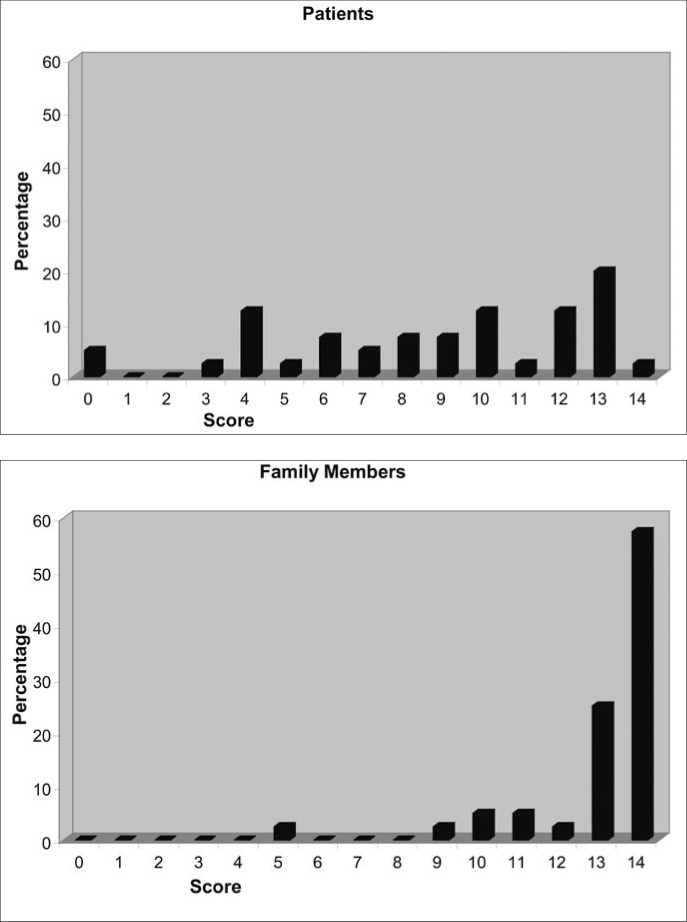
Histograms of the scores for the Schedule for Assessment of Insight (versions for patients and family members) among 40 patients and 40 family members in a psychiatric service in São Paulo, Brazil. t = −6.29; p < 0.001.

The mean SAI score was 8.75 (95% CI: 7.52 to 9.98) for the patients and 13.0 (95% CI: 12.42 to 13.58) for the family members. The histograms of scores for the two groups are shown in Figure 1. Family members performed better in the total and partial SAI scores, as shown in Table 2. However, when the scores were correlated for each patient-family member pair, the only partial score that had a negative correlation was the relabeling of psychotic phenomena (Table 3).

**Table 2:** Mean and 95% confidence interval of total and partial scores for the Schedule for Assessment of Insight in 40 patients with schizophrenia and 40 family members in a psychiatric service in São Paulo

	Patients	Family members	t	p
Adherence (95% CI)	3.4 (3.0 — 3.7)	3.9 (3.8 — 4.0)	-2.95	p < 0.005
Recognition of illness (95% CI)	3.5 (2.8 — 4.2)	5.5 (5.2 — 5.8)	-5.57	p < 0.001
Relabeling of psychotic phenomena (95% CI)	1.9 (1.3 — 2.4)	3.6 (3.2 — 3.9)	-5.27	p < 0.001
Total (95% CI)	8.75 (7.5 — 9.9)	13 (12.4 — 13.6)	-6.29	p < 0.001

*Note: Maximum scores for adherence and relabeling of psychotic phenomena = 4, and for recognition of illness = 6.*

*CI = confidence interval.*

**Table 3:** Correlation of the components of insight between 40 patients with schizophrenia and 40 family members (Spearman Rho test) in a psychiatric service in São Paulo

	Adherence (P)	Recognition of illness (P)	Relabeling of psychotic phenomena (P)	Total (P)
Adherence (F)	0.410†	0.278	0.299	0.365*
Recognition of illness (F)	0.214	0.422†	0.375*	0.479†
Relabeling of psychotic phenomena (F)	0.289	0.203	- 0.14	0.163
Total (F)	0.288	0.407†	0.248	0.401*

*Note: (F) = family members, (P) = patients*,

* *p < 0.05*,

† *p < 0.01.*

## DISCUSSION

The lack of relationship between insight and the demographic variables of age, sex and number of years of education is in agreement with many previous studies.^12,18,21,47^ However, this finding is not unanimous. There might be better insight among male patients^5^ and among patients with a higher educational level.^48^

Most studies have not found associations between insight and variables such as age of onset of illness, duration of illness and number of hospitalizations over lifetime.^18,21,49^ Others, however, have found better insight among patients with more hospitalizations,^50^ longer duration of illness^48^ and lower age at onset of illness.^44^

The fact that patients under psychological treatment had a better insight into the illness could be due to the psychoeducation provided by psychotherapy. Another explanation could be the reduction in the denial of the illness that would play a role in insight impairment. There may also be a bias, because patients with better insight would be more amenable to psychotherapy.

Family members scored significantly higher in all the components of the scale and in the overall score. The lack of correlation between the scores of family members and patients in the ability to relabel psychotic phenomena as abnormal can possibly be understood as the effect of stronger influence of cultural factors on the first two components of insight, namely adherence to treatment and recognition of illness. On the other hand, the relabeling of psychotic phenomena may be more influenced by psychopathology. This has also been suggested by Johnson and Orrell.^30^ Nevertheless, alternative explanations for this exist. The presence of schizoid personality traits is more common among family members of psychotic patients, and people with these traits could have more difficulty in identifying some symptoms of schizophrenia as such, because such symptoms may resemble these individuals’ traits.^51^ In addition, family members of psychotic patients also present more neuropsychological alterations than do control subjects.^52^ The presence of such psychopathological and neuropsychological characteristics among family members could account for the greater difficulty in identifying schizophrenia symptoms when they are present in other members of the family.

We should stress that the adapted scale used for family members did not prove to be a good instrument for this group. The scores obtained exhibited a concentration of high values, with little variation (*ceiling effect*), which made it impossible to properly discriminate the insight shown by this group. A similar finding has been reported in relation to another scale, the SUMD.^42^

### Limitations

Family members were not assessed for personality traits and neuropsychological deficits that could have influenced their ability to recognize schizophrenia symptoms among their relatives. With regard to the possibility of generalizing the results from this study, there was a selection bias, considering that the sample was recruited within a clinical setting. Such a sample would thus be more prone to have insight into illnesses, either as a factor influencing the seeking of treatment, or as an effect from the treatment. Further research evaluating patients and members of their families before their first contact with the treatment system could avoid such bias. Specific tools and methods have been developed to allow the diagnosis of psychiatric disorder in the general population by lay interviewers, i.e. including both those undergoing treatment and those who are not.^53^

## CONCLUSION

Since patients and members of their families share the same cultural environment, the significant difference regarding their insight can possibly be better explained by disease factors. Different degrees of insight are not equally influenced by disease and sociocultural factors. The recognition of illness seems to be more strongly influenced by sociocultural factors than is the ability to relabel psychotic phenomena as abnormal.
